# Comprehensive metabolomics analysis based on UPLC-Q/TOF-MS^E^ and the anti-COPD effect of different parts of *Celastrus orbiculatus* Thunb.

**DOI:** 10.1039/c9ra09965d

**Published:** 2020-02-26

**Authors:** Na Yang, Han Wang, Hongqiang Lin, Junli Liu, Baisong Zhou, Xiaoling Chen, Cuizhu Wang, Jinping Liu, Pingya Li

**Affiliations:** School of Pharmaceutical Sciences, Jilin University Fujin Road 126 Changchun 130021 Jilin China liujp@jlu.edu.cn lipy@jlu.edu.cn +86-431-85619803; Research Center of Natural Drug, Jilin University Changchun 130021 Jilin China

## Abstract

The root, stem and leaf of *Celastrus orbiculatus* Thunb. (COT) have all been used as Chinese folk medicine. Aiming at revealing the secondary metabolites and screening the anti-COPD effect of COT, the comprehensive phytochemical and bioassay studies were performed. Based on the ultra-high performance liquid chromatography combined with quadrupole time-of-flight mass spectrometry (UPLC-Q/TOF-MS^E^), the screening analysis of components in COT was conducted with the UNIFI platform, the metabolomics of the three parts were analyzed with multivariate statistical analysis. Cigarette smoke extract (CSE)-stimulated inflammatory model in A549 cells was used to investigate the biological effect of the three parts. A total of 120 compounds were identified or tentatively characterized from COT. Metabolomics analysis showed that the three parts of COT were differentiated, and there were 13, 8 and 5 potential chemical markers discovered from root, stem and leaf, respectively. Five robust chemical markers with high responses could be used for further quality control in different parts of COT. The root, stem and leaf of COT could evidently reduce the levels of pro-inflammatory factors in a dose-dependent way within a certain concentration range. The stem part had a stronger anti-COPD effect than root and leaf parts. This study clarified the structural diversity of secondary metabolites and the various patterns in different parts of COT, and provided a theoretical basis for further utilization and development of COT.

## Abstract 2

The root, stem and leaf of *Celastrus orbiculatus* Thunb. (COT) have all been used as Chinese folk medicine.

## Graphical abstract

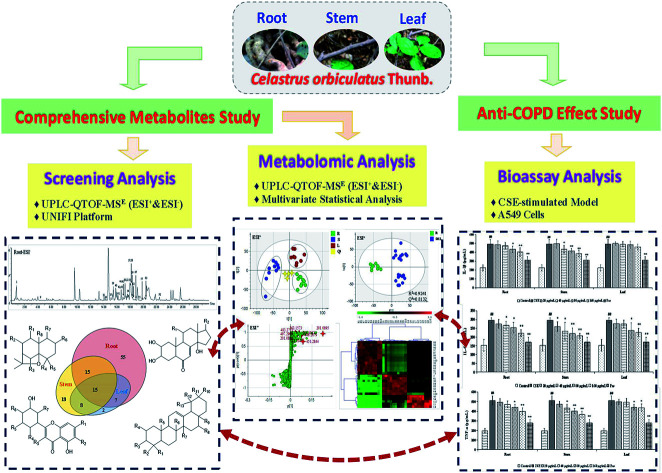

## 1. Introduction

*Celastrus orbiculatus* Thunb*.* (COT), belonging to the family Celastraceae and the genus *Celastrus* L., is widely distributed throughout China.^1^ The parts including root, stem and leaf all could been used as Chinese folk medicine to treat rheumatoid arthritis, vomiting, abdominal pain, and snakebites.^2,3^ The root of COT was reported to possess anti-tumor,^4^ antiviral,^5^ bacteriostatic^6^ and lipid-lowering^1^ activities, and about 50 compounds, including triterpenes, sesquiterpenoids, steroids and organic acids, were isolated from the root or root bark.^3,7–9^ The stem was also reported to have anti-inflammation,^10^ anti-cancer^11^ and fatty liver amelioration^12^ effects, and nearly 100 compounds including triterpenes, diterpenoids, steroids, flavonoids, phenolics and benzoquinone were isolated.^13–16^ For the leaf part, previous studies have found that the extract of it had insecticidal effect and hypoglycemic effect,^17^ while only a few flavonoids were isolated.^18^ It was revealed that there were significant variation for the contents of celastrol or total alkaloids in different parts of COT.^19,20^ Along with continuous expansion of the folk and clinical application of COT, an in-depth study on the chemical constituents in different parts of COT has attracted more and more attention. However, the comprehensive comparative study on the chemical composition between root, stem and leaf parts of COT has not been reported so far.

Recently, the UPLC-Q/TOF-MS method has been innovatively used for screening and identifying chemical components in herbal medicines and traditional Chinese medicine. And the global profiling of various metabolites were reported. As part of these research works, we reported this method to detect some natural products including *Platycodon grandiflorum* and Ginseng root.^21,22^ Our research results showed that this method is high throughput, comprehensive, simple and efficient. As far as we know, the UPLC-Q/TOF-MS method has not been reported to identify the components in COT. So, the study in this paper comparatively analyzes the phytochemicals of root, stem and leaf parts of COT by using the UPLC-Q/TOF-MS method for the first time and finds out the similarities and differences between them.

Chronic Obstructive Pulmonary Disease (COPD), predicted to rank as the third leading cause of death in the world,^23^ is mainly caused by significant exposure to harmful gases or particles.^24^ Cigarette smoking was the leading environmental risk factor for COPD around the world, and cigarette smokers were more likely to develop respiratory symptoms and had a higher COPD mortality rate. Along with the progressive lung inflammation, some pro-inflammatory mediators such as IL-1β, IL-6 and TNF-α participated in the occurrence and development of COPD.^25^ Although the COT had been used in treating various inflammatory diseases, the effect on the cigarette smoke extract (CSE)-induced inflammatory reaction has not been reported so far.

In the present study, the main medicinal parts of COT (root, stem and leaf) were chosen as the test sample. On one hand, the similarities and differences of phytochemicals in three parts were analyzed by using UNIFI platform and untargeted metabolomics based on UPLC-Q/TOF-MS^E^. The components and potential chemical markers to profile diverse classifications of metabolites of three parts were investigated. On the other hand, the effects on CSE-induced inflammatory reaction of these three parts were explored in A549 cells. The anti-COPD activity of different parts was preliminarily discussed. This comprehensive study could reveal the structural diversity of secondary metabolites and the different patterns of main medicinal parts of COT, and provide the data for further clinical application in anti-COPD. The study on the phytochemistry and the pharmacological activity of various parts were both significantly valuable to the research and development of COT.

## 2. Experiment

### 2.1. Materials and reagents

A total of 10 batches of fresh COT were collected from different growth areas in China (Table 1). All herbs were authenticated by the authors according to Hunan Province Local Standard for Traditional Chinese Medicine (2009 edition) for “*Celastrus orbiculatus* Thunb.”. The corresponding specimens had been deposited in the Research Center of Natural Drug, Jilin University, China.

**Table 1:** The detail information of the collected COT samples

No.	Source	Collection time
COT 01	Yichun city, Heilongjiang province, China	12th August, 2017
COT 02	Changchun city, Jilin province, China	3rd August, 2017
COT 03	Huhhot city, Inner Mongolia autonomous region, China	10th August, 2017
COT 04	Lanzhou city, Gansu province, China	13th August, 2017
COT 05	Taian city, Shandong province, China	5th July, 2017
COT 06	Xi'an city, Shanxi province, China	14th July, 2017
COT 07	Zhengzhou city, Henan province, China	15th July, 2017
COT 08	Chengdu city, Sichuan province, China	7th July, 2017
COT 09	Changsha city, Hunan province, China	27th July, 2017
COT 10	Jinhua city, Zhejiang province, China	2nd July, 2017

Methanol and acetonitrile were of LC/MS grade purchased from Fisher Chemical Company. Formic acid was bought from Sigma-Aldrich Company, St. Louis, MO, USA. Deionized water was purified by Millipore water purification system (Millipore, Billerica, MA, USA). All other chemicals were analytically pure. Cigarettes for bioassay analysis were Xiongshi cigarette (China Tobacco Zhejiang Industrial Co., Ltd, Hangzhou, China), each cigarette contained 11 mg of tar, 0.7 mg of nicotine, and 13 mg of carbon monoxide. Human lung carcinoma A549 cells were obtained from the Department of Pathogen Biology, Basic Medical College, Jilin University. ELISA kits were bought from Nanjing Jiancheng Bio-engineering Institute.

### 2.2. Sample preparation of three parts of COT

Took the whole fresh COT, and separated the root, stem and leaf part respectively to get 30 test samples including root part (R1–R10) samples, stem part (S1–S10) samples and leaf part (L1–L10) samples. The aforementioned parts were air-dried, grinded and sieved with Chinese National Standard Sieve No. 3, R40/3 series to obtain the homogeneous powder respectively. Each powder was weighted (2.0 g) accurately and extracted thrice (3 hours per time) with 100 mL of 80% methanol at 80 °C, cooled, filtered, collected and combined the filtrate of each sample, concentrated and evaporated to dryness.

For metabonomics analysis, each residue (all approximately 2.0 mg) was dissolved in 1.0 mL of 80% methanol respectively, after being filtered with a syringe filter (0.22 μm), 30 test solutions (R_M1_–R_M10_, S_M1_–S_M10_ and L_M1_–L_M10_) were obtained, which was injected into the UPLC system directly. Furthermore, to ensure the suitability consistency and the stability of MS analysis, a sample for quality control (QC) was prepared by pooling 20 μL from every test solution, namely containing all of the constituents in this analysis.

For screening analysis, the test solutions of root part (R_S_), stem part (S_S_) and leaf part (L_S_) were prepared by pooling 100 μL from R_M1_–R_M10_, S_M1_–S_M10_ and L_M1_–L_M10_ solutions, respectively.

For bioassay analysis, the test samples (R_bio_, S_bio_ and L_bio_) of root part, stem part and leaf part were prepared by combining each residue of R_1_–R_10_, S_1_–S_10_ and L_1_–L_10_, respectively. Then, R_bio_, S_bio_ and L_bio_ were dissolved in water at the concentration of 3.2 mg mL^−1^ to get the stock solutions stored in 4 °C.

### 2.3. Ultra-high performance liquid chromatography combined with quadrupole time-of-flight tandem mass spectrometry (UPLC-Q/TOF-MS^E^)

The separation and detection of components were performed on the UPLC system combined with Xevo G2-XS Q/TOF mass spectrometer (Waters Co., Milford, MA, USA) with an electrospray ionization (ESI) interface.

The ACQUITY UPLC BEH C18 column (100 mm × 2.1 mm, 1.7 μm) was bought from Waters Corporation (Milford, MA, USA). The moving phrase was consisted of eluent A (0.1% methanoic acid in water, v/v) and eluent B (0.1% methanoic acid in acetonitrile, v/v) in a liner gradient program (0–2 min, 10% B; 2–26 min, 10 → 90% B; 26–28 min, 90% B; 28–28.1 min, 90 → 10% B; 28.1–40 min, 10% B) with a flow rate of 0.4 mL min^−1^. Set the temperature of column and the sample manager at 30 °C and 15 °C, respectively. 10% and 90% acetonitrile in aqueous solution were used as weak and strong wash solvents respectively.

The optimized MS parameters were as follows: source temperature (150 °C), desolvation temperature (400 °C), cone voltage (40 V), capillary voltage at 2.6 kV (ESI^+^) and 2.2 kV (ESI^−^), cone gas flow (50 L h^−1^) and desolvation gas flow (800 L h^−1^). MS^E^ mode was chosen with low energy of 6 V and high energy of 20–40 V.^26,27^ The mass spectrometer was calibrated with sodium formate in the range of 100 to 1200 Da in order to ensure the mass reproducibility and accuracy. Leucine enkephalin (*m*/*z* 556.2771 in ESI^+^ and 554.2615 in ESI^−^) was used as external reference for Lock Spray™ injected at a constant flow of 10 μL min^−1^. The QC sample was injected randomly 4 times throughout the whole work list. All of the volume injection of the samples and QC was 5 μL per run. During data acquisition, the data for screening analysis was performed in MS^E^ continuum mode, the data for metabolomics analysis was performed in MS^E^ centroid mode. Data recording was performed on MassLynx V4.1 workstation (Waters, Manchester, UK).

### 2.4. Screening analysis of components in three parts of COT by UNIFI platform

UNIFI 1.7.0 software (Waters, Manchester, UK) was used for data analysis.^28,29^

Firstly, in addition to the internal Traditional Medicine Library on UNIFI platform, the chemical constituent investigation was conducted. As the result, a self-built database of chemical compounds isolated from the genus of *Celastrus* L. was established by searching the online databases including Web of Science, Medline, PubMed, ChemSpider and China National Knowledge Infrastructure (CNKI). The compound name, molecular formula and chemical structure of components were obtained in the database.

Secondly, the raw data obtained from Masslynx workstation were compressed by Waters Compression and Archival Tool v1.10, then were imported into the UNIFI software.

Thirdly, the compressed data were processed by the streamlined work flow of UNIFI software in order to quickly identify the chemical compounds which were matched the criteria with Traditional Medicine Library and self-built database. The main parameters of processed method were as follow: 2D peak detection was set to 200 as the minimum peak area. In the 3D peak detection, the peak intensity of high energy and low energy was taken more than 200 and 1000 times as the parameter respectively. Selected +H and +Na as positive adducts and +COOH and –H as negative adducts. Leucine enkephalin was used as reference compound in order to get exact mass accuracy, with [M + H]^+^ 556.2766 for positive ion and [M − H]^−^ 554.2620 for negative ion. As a result, the comprehensive chemical constituents screening list was accomplished.

Finally, a filter was set to refine the results, with the mass error between −5 and 5 ppm and response value over 5000. Each compound was verified by compared with the characteristic MS fragmentation patterns reported in literature or the retention time of the reference substances.

### 2.5. Metabolomics analysis of three parts of COT

The raw data acquired by Masslynx workstation was processed on MakerLynx XS V4.1 software (Waters, Milford, CT, USA). Firstly, the raw data were processed with alignment, deconvolution, and data reduction, *etc*. The main parameters of the process method were as follows: retention time (0–28 min), retention time window (0.20), mass (100–1200 Da), mass tolerance (0.10), mass window (0.10), minimum intensity (5%), marker intensity threshold (2000 counts) and noise elimination (level 6). As a result, the list with mass and retention time corresponded to the responses based on all the detected peaks from each data file were shown in Extended Statistics (XS) Viewer. Secondly, multivariate statistical analysis, both principle component analysis (PCA) and orthogonal projections to latent structures discriminant analysis (OPLS-DA), were performed on the MakerLynx software to analyze the resulting data.

PCA, a classical unsupervised low dimensional pattern recognition model, was used to show pattern recognition and maximum variation, and the overview and classification were obtained. OPLS-DA was used to obtain the maximum separation between two different groups. *S*-plots, which could provide visualization of the OPLS-DA predictive results, were created to explore the potential chemical markers which contributed to the differences.

Meanwhile, metabolites with VIP value > 4.0 and *p*-value < 0.001 were considered as potential chemical markers.^30,31^ Futhermore, permutation test was also performed to provide a reference distribution with the *R*^2^/*Q*^2^ values to indicate statistical significance. Finally, the analysis results were shown in Simca 15.0 software (Umetrics, Malmö, Sweden).

### 2.6. Bioassay analysis of three parts of COT

#### 2.6.1 Preparation of cigarette smoke extract

The preparation of the cigarette smoke extract (CSE) was basically the same as previous reports.^32^ Put the smoke from one cigarette into 20 mL culture medium (300 s per cigarette). The CSE solution was incubated at 37 °C for 30 min after being filtered with a 0.22 μm sterile filter. The CSE solution was prepared freshly and was used within 30 min. This prepared CSE solution was considered to have the highest concentration (100%).

#### 2.6.2 Cell viability assay

The final concentrations (20.0, 40.0, 80.0, 160.0, 320.0 μg mL^−1^) of each test samples (R_bio_, S_bio_ and L_bio_) were acquired by diluting the stock solutions with Dulbecco's Modified Eagle Medium (DMEM). A549 cells were cultured in 96-well plates at a density of 5 × 10^5^ cells per well treated with CSE (0%, 5%, 10%, 20%, 30% and 40%) for 18 h, or treated with R_bio_, S_bio_ and L_bio_ solutions (0.0, 20.0, 40.0, 80.0, 160.0, 320.0 μg mL^−1^) for 24 h. The growth-inhibition effect of CSE and the effect of the drugs on viability of A549 cells were evaluated by MTT assay.

#### 2.6.3 Drug treatment

For all groups, A549 cells were cultured in 96-well plates at a density of 5 × 10^5^ cells per mL for 18 h. In CSE group, the cells were treated with a certain dose of CSE without drug intervened. In drug groups, the cells were treated with both CSE and drugs (R_bio_, S_bio_ or L_bio_). In control group, A549 cells were cultured normally without the CSE or drugs. In positive group, the cells were treated with both CSE and dexamethasone (5 μg mL^−1^).

#### 2.6.4 Enzyme-linked immunosorbent assay

The contents of IL-1β, IL-6 and TNF-α in the cell culture supernatant were determined with ELISA kits. All procedures were performed according to the manufacturer's instructions.

#### 2.6.5 Statistical analysis

Statistical analysis was performed on Graphpad Prism 6.0 software (CA, USA). The results were expressed as mean ± SD. Two tailed test or a one-way analysis of variance (ANOVA) was used to calculate statistical significant difference (*p* < 0.05).

## 3. Results and discussion

### 3.1. Screening analysis of components of three parts of COT

**Fig. 1:**
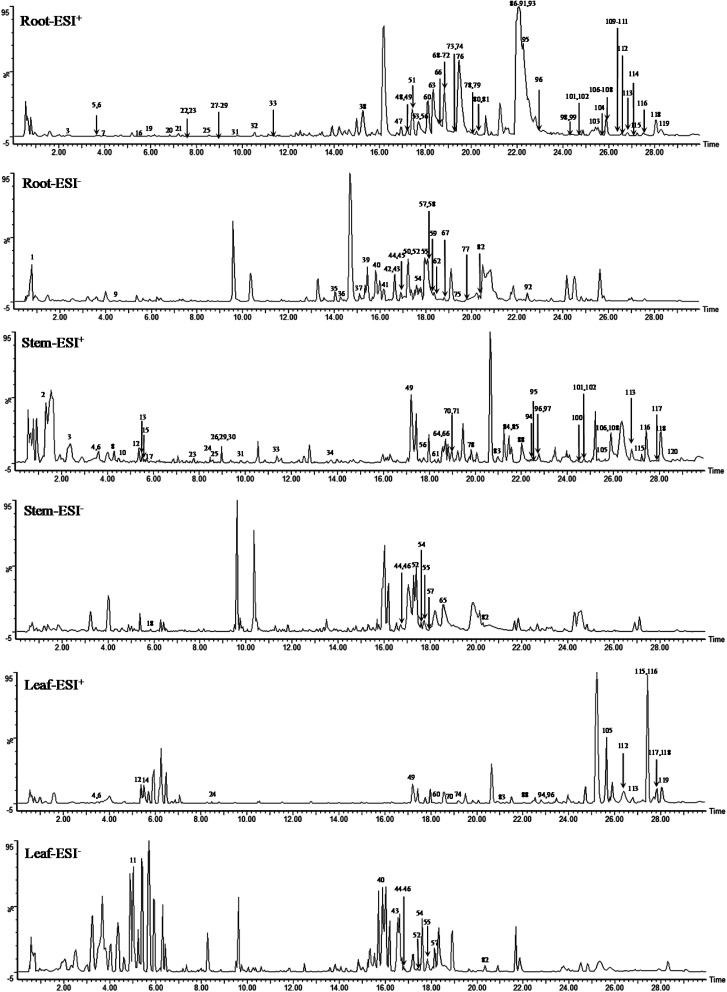
The base peak intensity (BPI) chromatograms in root, stem and leaf of COT in ESI^+^ and ESI^−^.

**Fig. 2:**
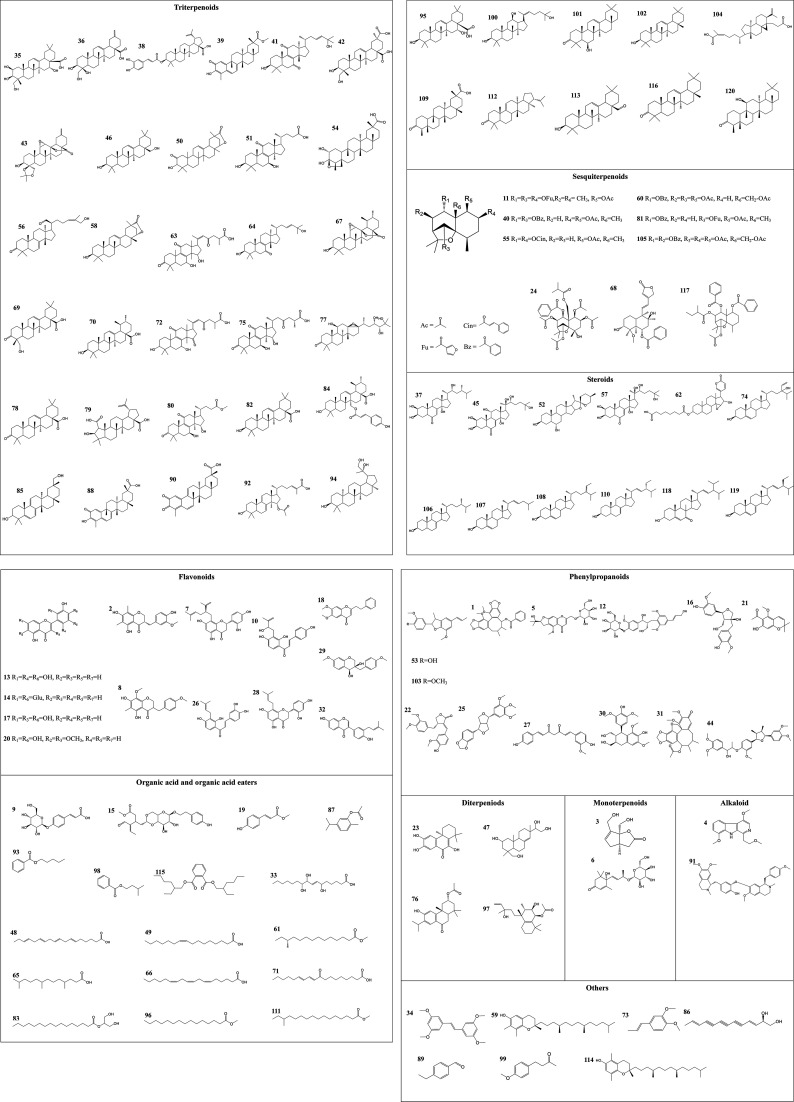
Chemical structures of compounds identified in the root, stem and leaf of COT.

**Fig. 3:**
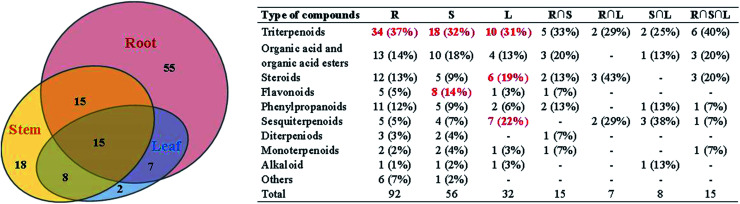
The numbers (% of the total identified components in each part) and structural types of compounds identified from root, stem and leaf of COT. R: the root of *Celastrus orbiculatus* Thunb.; S: the stem of *Celastrus orbiculatus* Thunb.; L: the leaf of *Celastrus orbiculatus* Thunb.

A total of 120 compounds, including 91 in ESI^+^ mode and 29 in ESI^−^ mode, were identified or tentatively characterized from three parts of COT (Table 2). The base peak intensity (BPI) chromatograms were shown in Fig. 1. The chemical structures were shown in Fig. 2, the results showed that COT was rich in natural components with various structural patterns. On one hand, according to the reference, there were nearly 50, 100, 10 compounds were reported from the root, stem, leaf parts of COT, respectively. While in this study, there were 92, 56 and 32 components were identified or tentatively characterized from root part, stem part and leaf part of COT, respectively. And most of the components were identified from COT for the first time. Various kinds of structures, including triterpeniods, sesquiterpenoids, steroids, flavonoids, organic acid and organic acid esters, phenylpropanoids, diterpeniods, monoterpenoids, alkaloid and others, were contained in each part of COT. The numbers (% of the total identified components in each part) and structural types of compounds identified from root, stem and leaf of COT were shown in Fig. 3. There were 34, 18 and 10 triterpeniods identified from root, stem and leaf, respectively, accounted for 37%, 32% and 31% of the total components in each part. So, it was concluded that triterpeniods were the major constituents in three parts of COT. Moreover, according to each percentage, the root part of COT was also rich in organic acid and organic acid esters, steroids and phenylpropanoids. The stem part was also rich in organic acid and organic acid esters, and flavonoids. The leaf part was also rich in steroids, and sesquiterpenoids. The percentage of flavonoids in the total identified components in stem was higher than the percentages in root part or in leaf part. The percentages of sesquiterpeniods and steroids in the total identified components in leaf was higher than the percentages in root part or in stem part. On the other hand, the shared components (30 for root and stem, 22 for root and leaf, 23 for stem and leaf, 15 for root, stem and leaf) were also found in our study. As shown in Fig. 3, the structures of shared components were various, while triterpeniods held the majority. Celastrol, one of the triterpeniods, was shown to distribute in root, stem and leaf, which was consistent with the ref. 19. So our research work could provide the scientific data to clarify the chemical composition of COT, particularly for the root and the leaf parts.

**Table 2:** Compounds identified from the root, stem and leaf of COT by UPLC-QTOF-MS^E^^a^

No.	*t*_R_ (min)	Formula	Calculated mass (Da)	Theoretical mass (Da)	Mass error (ppm)	MS^E^ fragmentation	Identification	Source	Ref.
1	0.59	C_29_H_28_O_8_	504.1828	504.1843	−2.7	549.1810 [M + H − COO]^−^, 382.1400 [M − H − C_7_H_5_O_2_]^−^, 311.0857 [M − H − C_11_H_12_O_3_]^−^	Interiotherin A	R	33
2#	1.23	C_19_H_20_O_6_	344.1256	344.1260	−1.1	367.1486 [M + Na]^+^, 327.1185 [M + H − H_2_O]^+^, 315.0707 [M + H − 2 × CH_3_]^+^, 238.0591 [M + H − C_7_H_7_O]^+^, 224.0341 [M + H − C_8_H_9_O]^+^	5,7-Dihydroxy-6,8-dimethyl-3(*S*)-3-(3-methoxy-4′-hydroxybenzyl)chroman-4-one	S	—
3	2.43	C_9_H_12_O_4_	184.0728	184.0736	−4.4	185.0791 [M + H]^+^, 167.0584 [M + H − H_2_O]^+^, 136.0593 [M + H − H_2_O − CH_2_OH]^+^	Eucommidiol	R, S	34
4	3.60	C_16_H_18_N_2_O_3_	286.1328	286.1317	3.5	309.1220 [M + Na]^+^, 242.0841 [M + H − 3 × CH_3_]^+^, 225.0989 [M + H − 2 × OCH_3_]^+^	Picrasidine B	S, L	a
5	3.64	C_22_H_28_O_11_	468.1616	468.1632	−3.4	469.1660 [M + H]^+^, 290.1036 [M + H-Glu]^+^, 232.0691 [M + H − C_3_H_7_O-Glu]^+^	Cimicifuga glycoside	R	35
6	3.66	C_19_H_30_O_8_	386.1925	386.1941	−4.2	409.1811 [M + Na]^+^, 226.0124 [M + H − C_6_H_9_O_5_]^+^, 178.0837 [M + H − 2 × CH_3_-Glu]^+^, 190.1255 [M + H − H_2_O-Glu]^+^	Roseoside	R, S, L	36
7	3.97	C_25_H_28_O_6_	424.1901	424.1886	3.6	447.1812 [M + Na]^+^, 407.1226 [M + H − H_2_O]^+^, 303.0539 [M + H − C_9_H_14_]^+^, 176.0533 [M + H − H_2_O − C_9_H_14_ − C_6_H_5_O_2_]^+^	Norkurarinone	R	37
8	4.17	C_19_H_20_O_6_	344.1244	344.1260	−4.7	345.1316 [M + H]^+^, 222.0530 [M + H − CH_3_ − C_7_H_7_O]^+^, 153.0511 [M + H − C_11_H_12_O_3_]^+^	2,3-Dihydro-5,7-dihydroxy-8-methoxy-3-[(4-methoxyphenyl)methyl]-6-methyl-4*H*-1-benzopyran-4-one	S	38
9	4.44	C_15_H_18_O_8_	326.0993	326.1002	−2.9	325.0870 [M − H]^−^, 128.0426 [M − H − H_2_O-Glu]^−^, 100.0409 [M − H − HCOOH-Glu]^−^, 75.0570 [M − H − C_3_H_3_O_2_-Glu]^−^	Glucosido-*p*-coumaric acid	R	s
10	4.60	C_20_H_20_O_5_	340.1295	340.1311	−4.5	341.1355 [M + H]^+^, 256.0641 [M + H − C_5_H_9_O]^+^, 238.0692 [M + H − H_2_O − C_5_H_9_O]^+^, 193.0713 [M + H − C_9_H_7_O_2_]^+^	Psorachalcone A	S	—
11※	5.02	C_32_H_34_O_12_	610.2048	610.2050	−0.4	609.1446 [M − H]^−^, 541.2011 [M − H − O − C_2_H_3_O]^−^, 371.1898 [M − H − O − 2 × C_5_H_3_O_3_]^−^	Orbiculin I	L	—
12	5.28	C_27_H_36_O_13_	568.2139	568.2156	−2.8	591.2031 [M + Na]^+^, 359.1459 [M + H − CH_2_OH-Glu]^+^, 345.1251 [M + H − CH_3_ − C_11_H_13_O_4_]^+^	Citrusin B	S, L	s
13	5.39	C_15_H_10_O_7_	302.0415	302.0427	−4.1	303.0484 [M + H]^+^, 178.0320 [M + H − C_6_H_5_O_3_]^+^, 108.0286 [M + H − H_2_O − C_9_H_5_O_4_]^+^	Isoetin	S	s
14※	5.53	C_27_H_30_O_16_	610.1539	610.1534	0.9	611.1613 [M + H]^+^, 432.1040 [M + H-Glu]^+^, 253.0388 [M + H − 2 × Glu]^+^	Luteolin 7,4′-diglucoside	L	39
15	5.65	C_24_H_32_O_10_	480.2007	480.1996	2.3	503.1899 [M + Na]^+^, 386.1517 [M + H − 2 × H_2_O − COOCH_3_]^+^, 360.1426 [M + H − C_8_H_9_O]^+^, 191.0715 [M + H − C_8_H_9_O − C_9_H_13_O_3_]^+^	Ilexin L3	S	—
16	5.66	C_20_H_24_O_7_	376.1505	376.1522	−4.5	399.1388 [M + Na]^+^, 316.1217 [M + H − 2 × CH_3_ − CH_2_OH]^+^, 310.1320 [M + H − 2 × H_2_O − CH_3_O]^+^, 138.0597 [M + H − C_12_H_15_O_5_]^+^	Vladinol C	R	40
17	5.74	C_15_H_10_O_7_	302.0418	302.0427	−2.9	303.0491 [M + H]^+^, 285.0363 [M + H − H_2_O]^+^, 110.0281 [M + H − C_9_H_5_O_5_]^+^	Quercetin	S	b
18	5.87	C_19_H_18_O_4_	310.1193	310.1205	−3.8	309.1266 [M − H]^−^, 279.0821 [M − H − 2 × CH_3_]^−^, 278.0977 [M − H − OCH_3_]^−^, 173.0532 [M − H − OCH_3_ − C_8_H_9_]^−^, 104.0703 [M − H − C_11_H_9_O_4_]^−^	6,7-Dimethoxy-2-phenethylchromone	S	s
19	5.96	C_10_H_10_O_3_	178.0623	178.0630	−3.9	179.0687 [M + H]^+^, 164.0374 [M + H − CH_3_]^+^, 120.0491 [M + H − COOCH_3_]^+^, 86.0307 [M + H − C_6_H_5_O]^+^	Methyl-*p*-coumarate	R	41
20	6.75	C_17_H_14_O_8_	346.0688	346.0689	−0.1	347.0761 [M + H]^+^, 329.0629 [M + H − H_2_O]^+^, 298.0688 [M + H − H_2_O − OCH_3_]^+^, 219.0460 [M + H − H_2_O − C_6_H_5_O_2_]^+^	Aksilarin	R	s
21	7.22	C_14_H_16_O_4_	248.1040	248.1049	−3.5	249.1105 [M + H]^+^, 206.0877 [M + H − CH_3_CO]^+^, 193.1007 [M + H − C_3_H_4_O]^+^	Evodinnol	R	42
22	7.58	C_21_H_22_O_6_	370.1410	370.1416	−1.7	371.1483 [M + H]^+^, 220 [M + H − C_9_H_11_O_2_]^+^, 152 [M + H − C_12_H_11_O_4_]^+^	(+)-7,8-Didehydroarctigenin	R	43
23	7.73	C_17_H_20_O_4_	288.1360	288.1362	−0.5	311.1252 [M + Na]^+^, 274.1090 [M + H − CH_3_]^+^, 238.0842 [M + H − 2 × H_2_O − CH_3_]^+^	(+)-Celaphanol A	R, S	s
24	8.47	C_34_H_44_O_14_	676.2738	676.2731	1.0	677.2811 [M + H]^+^, 585.2390 [M + H − H_2_O − CH_3_ − C_2_H_3_O_2_]^+^, 556.2650 [M + H − C_7_H_5_O_2_]^+^, 441.1997 [M + H − 4 × C_2_H_3_O_2_]^+^	Celangulin IV	S, L	—
25	8.57	C_22_H_24_O_7_	400.1407	400.1522	−3.8	401.1559 [M + H]^+^, 383.0623 [M + H − H_2_O]^+^, 365.2118 [M + H − 2 × H_2_O]^+^, 234.0763 [M + H − C_9_H_11_O_3_]^+^	Aschantin	R, S	44
26	8.83	C_20_H_20_O_5_	340.1295	340.1311	−4.5	341.1355 [M + H]^+^, 326.0641 [M + H − CH_3_]^+^, 299.0562 [M + H − C_3_H_7_]^+^, 150.0562 [M + H − C_4_H_7_ − C_8_H_7_O_2_]^+^	Corylifol B	S	45
27	8.94	C_20_H_18_O_5_	338.1143	338.1154	−3.3	339.1209 [M + H]^+^, 218.0945 [M + H − C_8_H_7_O]^+^, 176.0735 [M + H − H_2_O − C_9_H_7_O_2_]^+^	Demethoxycurcumin	R	46
28	8.95	C_20_H_20_O_6_	356.1247	356.1260	−3.6	357.1320 [M + H]^+^, 245.0820 [M + H − CH_3_ − C_6_H_5_O_2_]^+^, 96.0346 [M + H − H_2_O − C_14_H_11_O_4_]^+^	Leachianone G	R	s
29	8.96	C_18_H_20_O_5_	316.1317	316.1311	1.8	339.1209 [M + Na]^+^, 251.0624 [M + H − 2 × H_2_O − 2 × CH_3_]^+^, 174.0900 [M + H − 2 × H_2_O − C_7_H_7_O]^+^	(3*R-cis*)-3,4-Dihydro-3,4-diol-7-methoxy-3-[(4-methoxyphenyl)methyl]-2*H*-1-benzopyran	R, S	—
30#	8.98	C_22_H_28_O_8_	420.1799	420.1784	3.4	443.1696 [M + Na]^+^, 205.0843 [M + H − 2 × CH_2_OH − C_8_H_9_O_3_]^+^, 167.0664 [M + H − C_13_H_18_O_5_]^+^	(+)-Lyoniresinol	S	47
31	9.81	C_24_H_26_O_8_	442.1611	442.1628	−3.8	443.1675 [M + H]^+^, 384.1466 [M + H − C_2_H_3_O_2_]^+^, 381.1268 [M + H − 2 × CH_3_O]^+^, 307.0731 [M + H − CH_3_ − 2 × CH_3_O − C_2_H_3_O_2_]^+^	Interiorin C	R, S	—
32	10.35	C_20_H_18_O_4_	322.1218	322.1205	4.0	323.1291 [M + H]^+^, 268.1004 [M + H − C_4_H_7_]^+^, 254.0834 [M + H − C_5_H_9_]^+^	Neobavaisoflavone	R	48
33	11.39	C_18_H_34_O_5_	330.2392	330.2406	−4.0	353.2284 [M + Na]^+^, 277.2129 [M + H − 3 × H_2_O]^+^, 150.1100 [M + H − 2 × H_2_O − C_8_H_17_O_2_]^+^, 81.0569 [M + H − H_2_O − C_4_H_7_O_2_ − C_8_H_17_O_2_]^+^	9-Octadecenoic acid	R, S	s
34	13.75	C_18_H_20_O_4_	300.1348	300.1362	−4.3	323.1240 [M + Na]^+^, 138.0556 [M + H − C_10_H_11_O_2_]^+^, 107.0465 [M + H − CH_3_O − C_10_H_11_O_2_]^+^	*trans*-3,3′,5,5′-Tetramethoxystilbene	S	—
35*	14.06	C_30_H_48_O_6_	504.3448	504.3451	−0.6	549.3417 [M + HCOO]^−^, 263.3423 [M − H − C_14_H_24_O_3_]^−^, 239.3239 [M − H − C_16_H_24_O_3_]^−^	Virgaureagenin G	R	49
36	14.33	C_28_H_42_O_5_	458.3053	458.3032	4.6	457.2981 [M − H]^−^, 344.3321 [M − H − 2 × H_2_O − HCOOH − CH_2_OH]^−^, 233.2957 [M − H − C_13_H_20_O_3_]^−^,	3β,4β,23-Trihydroxy-24,30-dinorolean-12,20(29)-dien-28-oic acid	R	50
37	15.19	C_28_H_46_O_5_	462.3349	462.3345	0.8	461.3276 [M − H]^−^, 425.3270 [M − H − 2 × H_2_O]^−^, 193.2396 [M − H − C_17_H_32_O_2_]^−^	Polyporusterone F	R	51
38	15.26	C_39_H_54_O_6_	618.3926	618.3920	1.0	641.3857 [M + Na]^+^, 533.3344 [M + H − C_3_H_4_ − HCOOH]^+^, 398.2486 [M − 2 × CH_3_ − C_3_H_4_ − C_8_H_7_O_3_]^+^	Lup-20(29)-en-28-oic-3β-yl caffeate	R	s
39*	15.47	C_30_H_40_O_4_	464.2934	464.2927	1.5	509.2551 [M + HCOO]^−^, 386.2693 [M − H − H_2_O − C_2_H_3_O_2_]^−^, 199.2228 [M − H − C_17_H_28_O_2_]^−^	Pristimerin	R	52
40※	15.84	C_33_H_38_O_9_	578.2535	578.2516	3.3	577.2683 [M − H]^−^, 561.2680 [M − H − O]^−^, 547.2187 [M − H − 2 × CH_3_]^−^, 534.2176 [M − H − C_2_H_3_O]^−^	Orbiculin A	R, L	s
41	16.28	C_30_H_46_O_4_	470.3399	470.3396	0.6	469.3326 [M − H]^−^, 454.3157 [M − H − CH_3_]^−^, 451.3303 [M − H − H_2_O]^−^, 342.3522 [M − H − C_8_H_15_O]^−^	11-Oxokansenonol	R	53
42	16.52	C_30_H_46_O_6_	502.3315	502.3294	4.1	547.3326 [M + HCOO]^−^, 455.3236 [M − H − HCOOH]^−^, 401.3505 [M − H − 3 × H_2_O − HCOOH]^−^	Esculentic acid	R	s
43	16.66	C_31_H_44_O_6_	512.3118	512.3138	−3.9	511.3045 [M − H]^−^, 495.2929 [M − H − O]^−^, 467.2412 [M − H − CO_2_]^−^, 233.2535 [M − H − C_17_H_26_O_3_]^−^	Paeonenolide G	R, L	54
44	16.79	C_32_H_40_O_8_	552.2717	552.2723	−1.1	597.2699 [M + HCOO]^−^, 487.2250 [M − H − H_2_O − CH_3_ − CH_3_O]^−^, 193.1065 [M − H − C_21_H_26_O_5_]^−^	Saucerneol A	R, S, L	55
45	16.80	C_27_H_44_O_8_	496.3055	496.3036	3.8	541.3050 [M + HCOO]^−^, 461.3026 [M − H − 2 × H_2_O]^−^, 378.2876 [M − H − C_6_H_13_O_2_]^−^	Turkesterone	R, L	56
46	16.81	C_30_H_50_O_2_	442.3907	442.3811	−0.9	441.3713 [M − H]^−^, 423.3286 [M − H − H_2_O]^−^, 233.3456 [M − H − C_14_H_24_O]^−^	Oleanolic alcohol	S, L	57
47	17.06	C_20_H_34_O_4_	338.2461	338.2457	1.1	361.2353 [M + Na]^+^, 267.2239 [M + H − 4 × H_2_O]^+^, 262.1673 [M + H − CH_3_ − 2 × CH_2_OH]^+^, 278.0124 [M + H − C_3_H_5_O_2_]^+^	Hallol	R	a
48	17.22	C_18_H_28_O_2_	276.2086	276.2089	−1.0	277.2141 [M + H]^+^, 248.2197 [M + H − C_2_H_5_]^+^, 222.1520 [M + H − C_4_H_7_]^+^, 67.0579 [M + H − C_2_H_5_ − C_11_H_17_O_2_]^+^	6,9,12,15-Octadecatetraenoic acid	R	58
49	17.23	C_16_H_30_O_2_	254.2249	254.2246	1.2	277.2141 [M + Na]^+^, 125.1160 [M + H − C_4_H_9_ − C_3_H_5_O_2_]^+^, 112.0804 [M + H − H_2_O − C_9_H_17_]^+^	Oleopalmitic acid	R, S, L	59
50*	17.25	C_30_H_44_O_4_	468.3225	468.3240	−3.2	467.2441 [M − H]^−^, 434.2905 [M − H − H_2_O − CH_3_]^−^, 423.3320 [M − H − CO_2_]^−^	3β-Hydroxy-2-oxoolean-12-ene-22,29-actone	R	—
51*	17.30	C_27_H_40_O_6_	460.2833	460.2825	1.7	483.2751 [M + Na]^+^, 361.2355 [M + H − C_6_H_12_O]^+^, 417.2282 [M + H − CO_2_]^+^, 319.2480 [M + H − C_8_H_14_O_2_]^+^	Lucidenic acid N	R	60
52#	17.38	C_27_H_44_O_4_	432.3223	432.3240	−3.8	431.2217 [M − H]^−^, 397.3428 [M − H − O − H_2_O]^−^, 395.3258 [M − H − 2 × H_2_O]^−^, 249.1019 [M − H − C_11_H_18_O_2_]^+^	Chlorogenin	R, S, L	a
53	17.56	C_20_H_22_O_4_	326.1515	326.1518	−0.8	327.1588 [M + H]^+^, 312.1295 [M + H − CH_3_]^+^, 256.1067 [M + H − 2 × CH_3_ − C_3_H_5_]^+^, 203.2237 [M + H − C_7_H_8_O_2_]^+^	Dehydrodiisoeugenol	R	61
54※	17.62	C_30_H_48_O_5_	488.3523	488.3502	4.4	487.2917 [M − H]^−^, 469.3240 [M − H − H_2_O]^−^, 433.3297 [M − H − 3 × H_2_O]^−^, 411.1033 [M − H − 2 × CH_3_ − HCOOH]^+^, 405.3029 [M − H − 2 × H_2_O − HCOOH]^−^	Orthosphenic acid	R, S, L	a
55	17.80	C_35_H_40_O_7_	572.2788	572.2774	2.4	571.2738 [M − H]^−^, 555.2826 [M − H − O]^−^, 331.2334 [M − H − O − C_6_H_5_ − C_9_H_7_O_2_]^−^	Celastrine B	R, S, L	—
56	17.84	C_30_H_46_O_3_	454.3445	454.3447	−0.5	455.3492 [M + H]^+^, 408.3435 [M + H − H_2_O − CHO]^+^, 325.2291 [M + H − CH_3_ − C_6_H_11_O]^+^	(13α,14β,17α,20*S*,24*Z*)-Lanosta-26-hydroxy-3-oxo-7,24-dien-21-al	R, S	a
57*	18.02	C_27_H_44_O_7_	480.3106	480.3087	4.0	479.2439 [M − H]^−^, 464.2903 [M − H − CH_3_]^−^, 461.2934 [M − H − H_2_O]^−^, 414.2779 [M − H − H_2_O − C_2_H_7_O]^−^, 202.0239 [M − H − C_11_H_22_O_3_]^+^	Viticosterone	R, S, L	62
58	18.11	C_30_H_46_O_3_	454.3464	454.3447	3.8	453.3414 [M − H]^−^, 435.3608 [M − H − H_2_O]^−^, 409.3319 [M − H − CO_2_]^−^, 376.3716 [M − H − H_2_O − CH_3_ − CO_2_]^−^, 223.2100 [M − H − C_15_H_18_O_2_]^−^	Wilforlide A	R	s
59	18.20	C_28_H_48_O_2_	416.3636	416.3654	−4.1	461.3618 [M + HCOO]^−^, 265.2591 [M − H − C_9_H_10_O_2_]^−^, 149.2309 [M − H − C_19_H_38_]^−^	γ-Tocopherol	R	63
60	18.24	C_30_H_38_O_11_	574.2423	574.2414	1.5	597.2315 [M + Na]^+^, 395.1897 [M + H − C_2_H_3_O_2_ − C_7_H_5_O_2_]^+^, 339.1724 [M + H − 4 × C_2_H_3_O_2_]^+^	Ejap 3	R, L	a
61#	18.38	C_16_H_32_O_2_	256.2393	256.2402	−3.7	279.2303 [M + Na]^+^, 199.1787 [M + H − C_4_H_9_]^+^, 69.0553 [M + H − 2 × CH_3_ − C_9_H_17_O_2_]^+^	Methyl (12*S*)-12-methyltetradecanoate	S	a
62	18.41	C_32_H_44_O_8_	556.3016	556.3036	−3.6	555.2910 [M − H]^−^, 463.2604 [M − H − H_2_O − CH_3_ − C_2_H_3_O_2_]^−^, 313.2285 [M − H − H_2_O − C_7_H_13_O_2_ − C_5_H_3_O_2_]^−^	14,15-Epoxy-14*H*-cyclopenta [*a*]phenanthrene, bufa-20,22-dienolide deriv.	R	—
63*	18.46	C_30_H_42_O_7_	514.2931	514.2931	0.1	515.3017 [M + H]^+^, 469.2859 [M + H − HCOOH]^+^, 417.2608 [M + H − C_6_H_10_O]^+^, 377.2297 [M + H − C_9_H_14_O]^+^	Ganoderenic acid A	R	60
64#	18.67	C_30_H_48_O_3_	456.3583	456.3604	−4.7	457.3675 [M + H]^+^, 439.3520 [M + H − H_2_O]^+^, 424.2688 [M + H − H_2_O − CH_3_]^+^, 330.2465 [M + H − C_8_H_15_O]^+^	Kansenonol	S	64
65	18.69	C_16_H_32_O_2_	256.2394	256.2402	−2.7	301.2376 [M + HCOO]^−^, 97.0991 [M − H − C_6_H_13_ − C_3_H_5_O_2_]^−^, 68.0554 [M − H − CH_3_ − C_8_H_17_ − C_2_H_3_O_2_]^−^	4,8,12-Trimethyltridecanoic acid	S	65
66	18.69	C_18_H_30_O_2_	278.2233	278.2246	−4.5	279.2292 [M + H]^+^, 261.2353 [M + H − H_2_O]^+^, 72.0554 [M + H − C_11_H_19_ − C_2_H_3_O_2_]^+^	Gamolenic acid	R, S	66
67	18.74	C_30_H_46_O_4_	470.3399	470.3396	0.6	469.3326 [M − H]^−^, 454.3157 [M − H − CH_3_]^−^, 451.3303 [M − H − H_2_O]^−^, 407.3726 [M − H − H_2_O − CO_2_]^−^, 261.4102 [M − H − C_14_H_24_O]^−^	3β-Hydroxy-11α,12α-epoxy-13β,28-ursolide	R	a
68	18.83	C_28_H_36_O_7_	484.2458	484.2461	−0.6	469.2597 [M − OH]^+^, 363.2350 [M + H − C_7_H_6_O_2_]^+^, 345.1852 [M + H − H_2_O − C_7_H_6_O_2_]^+^, 218.1708 [M + H − 2 × H_2_O − C_7_H_6_O_2_ − C_6_H_5_O_2_]^+^	Scutellone I	R	67
69	18.94	C_30_H_46_O_4_	470.3399	470.3396	0.6	471.3326 [M + H]^+^, 425.3157 [M + H − HCOOH]^+^, 289.3241 [M + H − C_11_H_18_O_2_]^+^, 249.3383 [M + H − C_14_H_22_O_2_]^+^	Hederagonic acid	R	68
70	18.97	C_30_H_48_O_3_	456.3606	456.3604	0.5	457.3678 [M + H]^+^, 439.2231 [M + H − H_2_O]^+^, 249.3437 [M + H − C_14_H_24_O]^+^, 203.1653 [M + H − C_14_H_24_O − HCOOH]^+^	Ursolic acid	R, S, L	69
71	19.00	C_18_H_30_O_3_	294.2184	294.2195	−3.6	317.2060 [M + Na]^+^, 277.2115 [M + H − H_2_O]^+^, 172.0863 [M + H − C_9_H_15_]^+^, 124.1081 [M + H − C_9_H_15_O_3_]^+^	9-Oxooctadeca-10,12-dienoic acid	R, S	70
72	19.13	C_30_H_42_O_7_	514.2931	514.2931	0.1	515.3004 [M + H]^+^, 454.2660 [M + H − CH_3_ − HCOOH]^+^, 415.2676 [M + H − C_6_H_12_O]^+^, 321.2393 [M + H − C_11_H_14_O_3_]^+^	Ganoderenic acid B	R	60
73*	19.30	C_11_H_14_O_2_	178.1001	178.0994	4.2	201.0886 [M + Na]^+^,164.0739 [M + H − CH_3_]^+^, 107.0589 [M + H − CH_3_O − C_3_H_5_]^+^, 76.0507 [M + H − 2 × CH_3_O − C_3_H_5_]^+^	Isohomogenol	R	71
74	19.35	C_29_H_48_O_2_	428.3675	428.3654	4.7	451.3568 [M + Na]^+^, 411.2449 [M + H − H_2_O]^+^, 274.2205 [M + H − C_10_H_19_O]^+^, 256.2260 [M + H − H_2_O − C_10_H_19_O]^+^	Seringosterol	R, L	72
75	19.46	C_30_H_44_O_7_	516.3080	516.3087	−1.3	515.3007 [M − H]^−^, 500.2865 [M − H − CH_3_]^−^, 497.3383 [M − H − H_2_O]^−^, 322.1767 [M − H − 2 × H_2_O − C_8_H_13_O_3_]^−^	Ganoderic acid A	R	73
76	19.72	C_22_H_30_O_4_	358.2132	358.2144	−3.3	381.2024 [M + Na]^+^, 316.2063 [M + H − C_2_H_3_O]^+^, 273.1648 [M + H − C_3_H_7_ − C_2_H_3_O_2_]^+^	(3*S*,4*aS*,10*aS*)-3-(Acetyloxy)-2,3,4,4*a*,10,10*a*-hexahydro-6-hydroxy-1,1,4*a*-trimethyl-7-(1-methylethyl)-9(1*H*)-phenanthrenone	R	s
77	19.73	C_30_H_50_O_6_	506.3632	506.3607	4.9	505.3559 [M − H]^−^, 43.3909 [M − H − 4 × H_2_O]^−^, 431.2834 [M − H − CH_3_ − C_3_H_7_O]^−^	13β,17β-Epoxyalisol A	R	s
78	19.89	C_30_H_46_O_3_	454.3438	454.3447	−2.0	455.3492 [M + H]^+^, 409.3435 [M + H − HCOOH]^+^, 391.1517 [M + H − H_2_O − HCOOH]^+^, 249.3317 [M + H − C_14_H_22_O]^+^	Oleanonic acid	R, S	74
79	20.05	C_30_H_46_O_5_	486.3334	486.3345	−2.4	487.3406 [M + H]^+^, 441.3344 [M + H − HCOOH]^+^, 413.3436 [M + H − H_2_O − CH_3_ − HCOOH]^+^, 395.3278 [M + H − 2 × HCOOH]^+^, 249.5358 [M + H − C_14_H_22_O_3_]^+^	Ceanothic acid	R	a
80*	20.24	C_28_H_42_O_6_	474.2986	474.2981	1.0	497.2908 [M + Na]^+^, 457.2342 [M + H − H_2_O]^+^, 411.2324 [M + H − H_2_O − CH_3_ − CH_3_O]^+^, 285.0399 [M + H − H_2_O − C_9_H_16_O_3_]^+^	Methyl lucidenate Q	R	75
81	20.36	C_29_H_34_O_8_	510.2256	510.2254	0.3	533.2148 [M + Na]^+^, 410.1760 [M + H − C_2_H_3_O − C_3_H_6_O]^+^, 268.1455 [M + H − C_2_H_3_O − C_7_H_5_O − C_5_H_3_O_2_]^+^	Orbiculin B	R	—
82	20.36	C_30_H_48_O_3_	456.3617	456.3604	3.0	455.3544 [M − H]^−^, 391.3440 [M − H − H_2_O − HCOOH]^−^, 149.3707 [M − H − H_2_O − C_18_H_27_O_2_]^−^	Oleanolic acid	R, S, L	74
83	21.14	C_19_H_38_O_4_	330.2770	330.2770	0.1	353.2632 [M + Na]^+^, 313.2160 [M + H − H_2_O]^+^, 300.0486 [M + H − CH_3_O]^+^, 240.0679 [M + H − C_3_H_7_O_3_]^+^	β-Monopalmitin	S, L	76
84#	21.46	C_39_H_54_O_6_	618.3949	618.3920	4.6	619.4002 [M + H]^+^, 565.4126 [M + H − 3 × H_2_O]^+^, 472.3557 [M + H − C_9_H_7_O_2_]^+^, 456.3443 [M + H − C_9_H_7_O_3_]^+^, 411.1356 [M + H − C_14_H_24_O]^+^	27-*p-E*-Coumaroyloxyursolic acid	S	77
85#	21.49	C_30_H_46_O_2_	438.3485	438.3498	−2.9	439.3559 [M + H]^+^, 421.3432 [M + H − H_2_O]^+^, 390.1126 [M + H − H_2_O − CH_3_O]^+^, 250.3443 [M + H − C_13_H_17_O]^+^	5-Dehydrokarounidiol	S	—
86*	21.72	C_13_H_12_O_2_	200.0834	200.0831	−3.3	201.0886 [M + H]^+^, 186.0597 [M + H − CH_3_]^+^, 165.0996 [M + H − 2 × H_2_O]^+^, 140.0533 [M + H − C_2_H_5_O_2_]^+^	Safynol	R	78
87*	22.03	C_12_H_16_O_2_	192.1157	192.1150	3.7	215.1045 [M + Na]^+^, 163.0490 [M + H − 2 × CH_3_]^+^, 150.1739 [M + H − C_3_H_7_]^+^, 135.2173 [M + H − CH_3_ − C_3_H_7_]^+^, 119.0695 [M + H − CH_3_ − C_2_H_3_O_2_]^+^, 91.0556 [M + H − C_3_H_7_ − C_2_H_3_O_2_]^+^	Carvacryl acetate	R	a
88*	22.04	C_29_H_38_O_4_	450.2766	450.2770	−0.9	451.2844 [M + H]^+^, 433.2591 [M + H − H_2_O]^+^, 405.2771 [M + H − HCOOH]^+^, 215.3621 [M + H − C_15_H_24_O_2_]^+^, 200.0917 [M + H − CH_3_ − C_15_H_24_O_2_]^+^	Celastrol	R, S, L	79
89	22.05	C_9_H_10_O	134.0737	134.0732	3.4	157.0632 [M + Na]^+^, 120.1173 [M + H − CH_3_]^+^, 106.0704 [M + H − C_2_H_5_]^+^, 77.0477 [M + H − C_2_H_5_ − CHO]^+^	4-Ethylbenzaldehyde	R	80
90*	22.22	C_29_H_36_O_4_	448.2609	448.2614	−1.0	449.2679 [M + H]^+^, 434.2548 [M + H − CH_3_]^+^, 403.2606 [M + H − HCOOH]^+^, 267.3315 [M + H − C_12_H_6_O_2_]^+^	25-(9→8)Abeo-24-nor-friedelan-2,3-dioxo-1(10),4,6,9(11)-tetraen-29-oic acid	R	—
91	22.46	C_38_H_44_N_2_O_6_	624.3200	624.3220	3.2	647.3113 [M + Na]^+^, 503 [M + H − C_8_H_9_O]^+^, 314.1395 [M + H − CH_3_ − C_18_H_16_NO_2_]^+^, 297 [M + H − C_7_H_7_O − C_11_H_17_O_2_N]^+^, 266.0790 [M + H − CH_3_ − C_8_H_9_O − C_13_H_18_NO_2_]^+^	Neferin	R	81
92	22.78	C_32_H_48_O_5_	512.3486	512.3502	−3.1	511.3413 [M − H]^−^, 493.2436 [M − H − H_2_O]^−^, 465.1023 [M − H − HCOOH]^+^, 434.2379 [M − H − H_2_O − C_2_H_3_O_2_]^−^, 370.2491 [M − H − C_8_H_13_O_2_]^−^	Ganoderic acid X	R	82
93	22.80	C_12_H_16_O_2_	192.1159	192.1150	4.2	215.1052 [M + Na]^+^, 178.0893 [M + H − CH_3_]^+^, 107.5147 [M − C_5_H_11_O]^+^, 78.0571 [M + H − C_6_H_11_O_2_]^+^	Amyl benzoate	R	s
94	22.81	C_30_H_52_O_3_	460.3920	460.3917	0.8	483.3813 [M + Na]^+^, 407.3593 [M + H − H_2_O]^+^, 368.3012 [M + H − H_2_O − C_3_H_7_O_2_]^+^, 253.0154 [M + H − C_14_H_24_O]^+^	Olibanumol H	S, L	83
95	22.82	C_30_H_48_O_4_	472.3543	472.3553	−2.1	473.3615 [M + H]^+^, 455.3483 [M + H − H_2_O]^+^, 427.3561 [M + H − HCOOH]^+^, 333.1527 [M + H − C_9_H_16_O]^+^, 290.0146 [M + H − CH_3_ − C_11_H_20_O]^+^	Echinocystic acid	R, S	84
96	22.83	C_16_H_32_O_2_	256.2390	256.2402	−4.5	279.2276 [M + Na]^+^, 155.1230 [M + H − C_5_H_11_ − CH_3_O]^+^, 85.0488 [M + H − C_10_H_21_ − OCH_3_]^+^	Methyl pentadecanoate	R, S, L	s
97	23.02	C_22_H_36_O_4_	364.2601	364.2614	−3.7	365.2665 [M + H]^+^, 266.2190 [M + H − C_6_H_11_O]^+^, 219.1749 [M + H − H_2_O − C_2_H_3_O_2_ − C_5_H_9_O]^+^	Vitetrifolin E	S	85
98	24.29	C_12_H_16_O_2_	192.1159	192.1150	4.2	215.1052 [M + Na]^+^, 164.0893 [M + H − CH_3_]^+^, 136.0587 [M + H − C_4_H_9_]^+^, 78.0571 [M + H − C_6_H_11_O_2_]^+^	Isopentyl benzoate	R	s
99	24.31	C_11_H_14_O_2_	178.1003	178.0994	4.7	201.0895 [M + Na]^+^, 147.0441 [M + H − CH_3_]^+^, 136.0491 [M + H − C_2_H_3_O]^+^, 122.0477 [M + H − C_3_H_5_O]^+^, 77.0146 [M + H − CH_3_O − C_4_H_7_O]^+^	Anisylacetone	R	86
100#	24.51	C_30_H_52_O_3_	460.3898	460.3917	−4.1	483.3682 [M + Na]^+^, 425.3325 [M + H − 2 × H_2_O]^+^, 336.2443 [M + H − C_9_H_17_]^+^, 295.0357 [M + H − C_12_H_22_]^+^	Neopanaxadiol	S	87
101	24.71	C_30_H_48_O_2_	440.3656	440.3654	0.3	441.3729 [M + H]^+^, 423.3611 [M + H − H_2_O]^+^, 259.1687 [M + H − C_11_H_18_O_2_]^+^, 219.0312 [M + H − C_14_H_22_O_2_]^+^	Daturanolone	R, S	88
102	24.94	C_30_H_48_O	424.3701	424.3705	−1.0	425.3774 [M + H]^+^, 407.3633 [M + H − H_2_O]^+^, 392.3459 [M + H − H_2_O − CH_3_]^+^, 218.1926 [M + H − C_14_H_23_O]^+^	Olean-9(11),12-dien-3β-ol	R, S	s
103*	25.48	C_21_H_24_O_4_	340.1672	340.1675	−0.8	363.1573 [M + Na]^+^, 189.0711 [M + H − CH_3_ − C_8_H_9_O_2_]^+^, 173.0939 [M + H − CH_3_O − C_8_H_9_O_2_]^+^, 107.0494 [M + H − CH_3_O − C_13_H_15_O_2_]^+^	Dehydrodiisoeugenol methyl ether	R	89
104	25.67	C_30_H_46_O_4_	470.3372	470.3396	−4.1	471.3445 [M + H]^+^, 456.2016 [M + H − CH_3_]^+^, 398.5107 [M + H − C_3_H_5_O_2_]^+^, 379.0317 [M + H − 2 × HCOOH]^+^, 312.2370 [M + H − H_2_O − C_8_H_13_O_2_]^+^, 234.4581 [M + H − CH_3_ − C_3_H_5_O_2_ − C_8_H_13_O_2_]^+^	Nigranoic acid	R	a
105	25.70	C_37_H_42_O_13_	694.2801	694.2778	3.6	695.2873 [M + H]^+^, 515.2201 [M + H − 3 × C_2_H_4_O_2_]^+^, 393.2228 [M + H − 3 × C_2_H_4_O_2_ − C_7_H_6_O_2_]^+^, 331.0167 [M + H − 2 × C_2_H_4_O_2_ − C_7_H_6_O_2_ − C_7_H_5_O_2_]^+^	Celahin D	S, L	s
106	25.91	C_28_H_48_O	400.3698	400.3705	−1.6	423.3591 [M + Na]^+^, 386.2638 [M + H − CH_3_]^+^, 326.2823 [M + H − H_2_O − CH_3_ − C_3_H_7_]^+^, 254.2266 [M + H − H_2_O − C_8_H_17_O]^+^, 213.2252 [M + H − H_2_O − C_11_H_22_O]^+^	Fungisterol	R, S	a
107	25.93	C_27_H_44_O	384.3390	384.3392	−0.6	385.3463 [M + H]^+^, 367.0417 [M + H − H_2_O]^+^, 256.2547 [M + H − H_2_O − C_8_H_15_]^+^, 215.2415 [M + H − H_2_O − C_11_H_20_]^+^	(*E*)-22-Dehydrocholesterol	R	90
108	25.96	C_29_H_48_O	412.3686	412.3705	−4.6	413.3746 [M + H]^+^, 260.2204 [M + H − CH_3_ − C_10_H_19_]^+^, 257.2151 [M + H − H_2_O − C_10_H_19_]^+^	28-Isofucosterol	R, S	a
109	26.22	C_30_H_48_O_3_	456.3588	456.3604	−3.5	457.3651 [M + H]^+^, 439.2142 [M + H − H_2_O]^+^, 411.3584 [M + H − HCOOH]^+^, 265.1812 [M + H − C_13_H_20_O]^+^	Maytenoic acid	R	s
110	26.28	C_29_H_48_O	412.3702	412.3705	−0.8	413.3775 [M + H]^+^, 395.3676 [M + H − H_2_O]^+^, 256.2275 [M + H − H_2_O − C_10_H_19_]^+^, 218.7218 [M + H − CH_3_ − C_13_H_24_]^+^	Chondryllasterol	R	91
111	26.31	C_18_H_36_O_2_	284.2724	284.2715	2.7	307.2616 [M + Na]^+^, 140.1312 [M + H − 2 × CH_3_ − C_6_H_11_O_2_]^+^, 70.0583 [M + H − 2 × CH_3_ − C_11_H_21_O_2_]^+^	Hexadecanoic acid	R	s
112	26.62	C_30_H_48_O	424.3686	424.3705	−4.4	425.3747 [M + H]^+^, 410.3584 [M + H − CH_3_]^+^, 367.3099 [M + H − CH_3_ − C_3_H_7_]^+^, 273.3120 [M + H − C_11_H_20_]^+^	Fernenone	R, L	92
113	26.83	C_30_H_48_O_2_	440.3656	440.3654	0.3	441.3729 [M + H]^+^, 423.3611 [M + H − H_2_O]^+^, 412.0147 [M + H − CHO]^+^, 233.1890 [M + H − C_14_H_24_O]^+^, 204.3167 [M + H − CHO − C_14_H_24_O]^+^	Oleanolic aldehyde	R, S, L	a
114	27.08	C_28_H_48_O_2_	416.3667	416.3654	2.9	439.3559 [M + Na]^+^, 192.1212 [M + H − C_16_H_33_]^+^, 151.0731 [M + H − C_19_H_38_]^+^	Cumotocopherol	R	93
115	27.25	C_24_H_38_O_4_	390.2752	390.2770	−4.3	413.2644 [M + Na]^+^, 179.0809 [M + H − C_2_H_5_ − C_4_H_9_ − C_8_H_17_O]^+^, 149.1469 [M + H − C_8_H_17_ − C_8_H_17_O]^+^, 77.0216 [M + H − 2 × C_9_H_17_O_2_]^+^	Fleximel	R, S, L	b
116	27.52	C_30_H_48_O	424.3709	424.3705	0.9	425.3782 [M + H]^+^, 410.3572 [M + H − CH_3_]^+^, 259.2141 [M + H − C_11_H_18_O]^+^, 221.1939 [M + H − C_14_H_20_O]^+^	β-Amyron	R, S, L	94
117※	27.84	C_36_H_44_O_9_	620.3003	620.2985	2.9	621.3083 [M + H]^+^, 562.2851 [M + H − C_2_H_3_O_2_]^+^, 461.2303 [M + H − C_2_H_3_O_2_ − C_5_H_9_O_2_]^+^, 291.2088 [M + H − C_2_H_5_ − C_2_H_3_O_2_ − 2 × C_7_H_5_O_2_]^+^	Celafolin D-3	S, L	s
118	27.88	C_30_H_48_O_2_	440.3656	440.3654	0.3	441.3729 [M + H]^+^, 423.3611 [M + H − H_2_O]^+^, 398.5359 [M + H − C_3_H_7_]^+^, 288.2288 [M + H − C_11_H_21_]^+^, 247.0117 [M + H − C_14_H_26_]^+^	Polasterol A	R, S, L	a
119	28.10	C_29_H_46_O	410.2535	410.2549	−3.4	411.3599 [M + H]^+^, 393.3696 [M + H − H_2_O]^+^, 272.2261 [M + H − C_10_H_19_]^+^, 231.2084 [M + H − C_13_H_24_]^+^	Corbisterin	R, L	95
120	28.49	C_30_H_50_O_2_	442.3813	442.3811	0.5	465.3705 [M + Na]^+^, 425.3647 [M + H − H_2_O]^+^, 291.3580 [M + H − C_10_H_16_O]^+^, 251.5133 [M + H − C_13_H_20_O]^+^	3-Oxo-11β-hydroxyfriedelane	S	96

a * Characteristic component in root; # characteristic component in stem; ※ characteristic component in leaf; a: compared with spectral data obtained from Wiley Subscription Services, Inc. (USA); b: compared with NIST Chemistry WebBook; s: compared with the reference compounds; R: the root of *Celastrus orbiculatus* Thunb.; S: the stem of *Celastrus orbiculatus* Thunb.; L: the leaf of *Celastrus orbiculatus* Thunb.

Although the study provided evidences to elucidate the chemical composition of COT, there were still some unresolved issues. For example, as shown in BPI chromatograms, there were some unidentified components. Further research should be carried on the identification of these unknown compounds.

### 3.2. Metabolomics analysis of three parts of COT

**Fig. 4:**
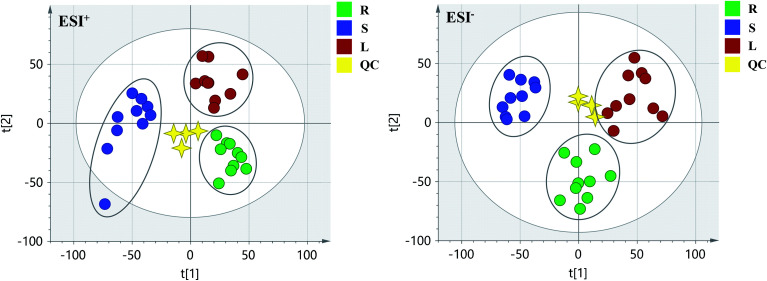
The PCA of root (R), stem (S), leaf (L) groups in ESI^+^ and ESI^−^. QC: quality control.

PCA score 2D plots in both ESI^+^ and ESI^−^ were established as shown in Fig. 4. The QC samples were clustered tightly and were in the middle of the three groups in PCA, which indicated the system had satisfactory stability. The samples from root of COT were clearly gathered together, which indicated there was a good similarity among them, and this phenomenon was also observed in stem and leaf of COT. Meanwhile, the root, stem and leaf groups were easily divided into three clusters, indicating that these three parts of COT could be differentiated in both ESI^+^ and ESI^−^.

**Fig. 5:**
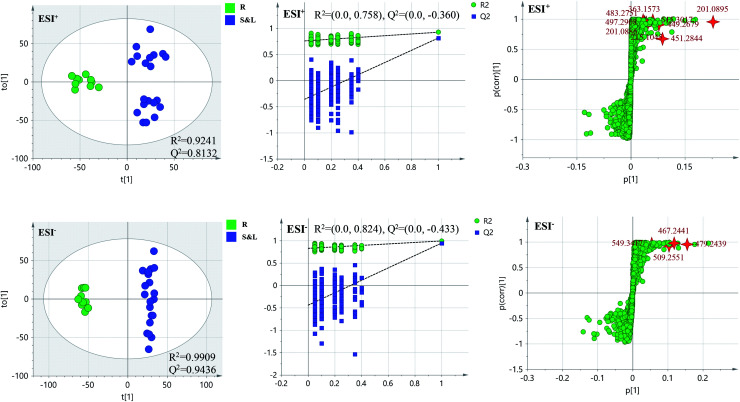
OPLS-DA plots/permutation tests/*S*-plots between R and S&L. R: the root of *Celastrus orbiculatus* Thunb.; S: the stem of *Celastrus orbiculatus* Thunb.; L: the leaf of *Celastrus orbiculatus* Thunb.

**Fig. 6:**
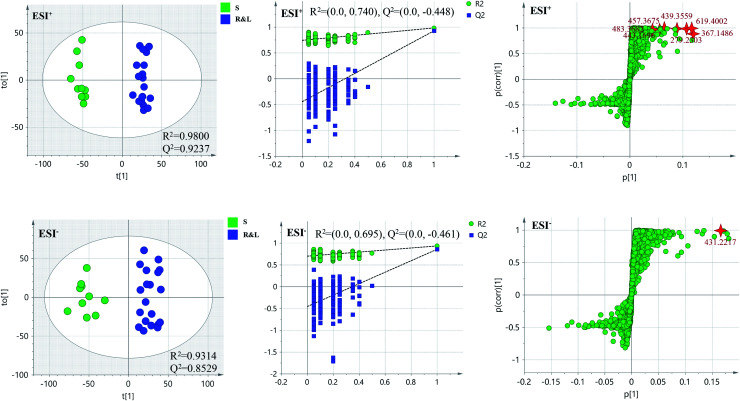
OPLS-DA plots/permutation tests/*S*-plots between S and R&L. R: the root of *Celastrus orbiculatus* Thunb.; S: the stem of *Celastrus orbiculatus* Thunb.; L: the leaf of *Celastrus orbiculatus* Thunb.

**Fig. 7:**
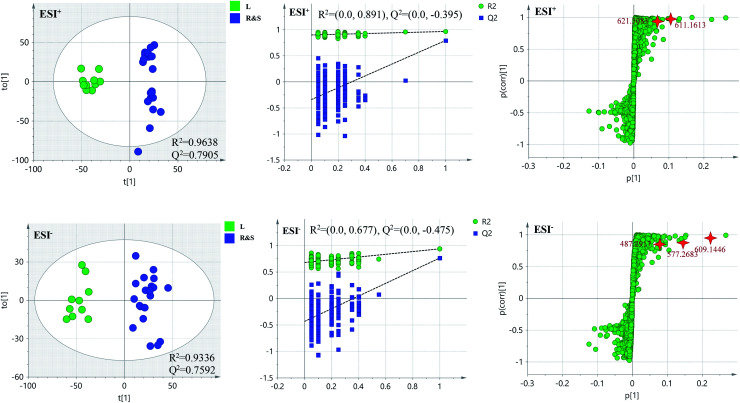
OPLS-DA plots/permutation tests/*S*-plots between L and R&S. R: the root of *Celastrus orbiculatus* Thunb.; S: the stem of *Celastrus orbiculatus* Thunb.; L: the leaf of *Celastrus orbiculatus* Thunb.

In order to further distinguish one part from the other two parts, OPLS-DA plots, *S*-plots, permutation tests, and VIP values were obtained to see which variables were responsible for sample separation^97^ (Fig. 5–7). In OPLS-DA plots, each spot represented a sample. From the perspective of OPLS-DA, one part was clearly separated from the other two parts. The parameters such as *R*^2^ and *Q*^2^ indicated the model had good ability of prediction and reliability in both ESI^+^ and ESI^−^ modes. The permutation plots showed the original point on the right was clearly higher than all *Q*^2^-values (blue) on the left, which indicated the original models were valid. To identify the metabolites contributing to the discrimination, *S*-plots were generated under OPLS-DA model. Each spot in *S*-plots represented a variable. The variables with VIP > 4 and *p <* 0.001 were considered as potential chemical markers. The possible molecular formula of the markers were calculated by high-accuracy quasi-molecular ion with mass error between ±5 ppm. A total of 26 robust known chemical markers (marked in Table 2) enabling the differentiation between one part with the other two parts were identified and marked in *S*-plots.

According to the reference, it was revealed that there was significant variation for the contents of celastrol or total alkaloids in different parts of COT. While in this study, there were 13, 8 and 5 potential chemical markers including celastrol discovered from root, stem and leaf, respectively. The markers in root including 8 triterpenoids (35, 39, 50, 51, 63, 80, 88, 90), 1 steroids (57), 1 organic acid esters (87), 1 phenylpropanoids (103) and 2 other compounds (73, 86). The markers in stem including 4 triterpenoids (64, 84, 85, 100), 1 flavonoids (2), 1 phenylpropanoids (30), 1 steroids (52) and 1 organic acid esters (61). The markers in leaf including 3 sesquiterpenoids (11, 40, 117), 1 flavonoids (14) and 1 triterpenoids (54). Additionally, among these potential chemical markers, the contents of 57 and 88 in root, 52 in stem, 40, 54 and 117 in leaf were much higher than in the other two parts (*p* < 0.001). While components 35, 39, 50, 51, 63, 73, 80, 86, 87, 90 and 103 were detected only in root, components 2, 30, 61, 64, 84, 85 and 100 were detected only in stem, components 11 and 14 were detected only in leaf part under the detect condition.

The detected result of 88 (celastrol) in our study, with much higher contents in root than in stem and leaf, was consistent with the ref. 19. While there were a few differences between our results and the references. In the present study, compound 39 (pristimerin) was only detected in root, and 11 (orbiculin I) was only detected in leaf. According to the reports, 39 was once isolated from stem^14^ though mainly from root,^98,99^ and 11 isolated from root.^3^ The reason was the concentrations of them were lower than the lowest detection limits. It was worth mentioning that some chemical markers with high responses in UPLC-MS, two triterpenoids (39 and 88) in root, one flavonoids (2) in stem and two sesquiterpenoids (11 and 40) in leaf, could be used for further quality control of three parts of COT respectively.

**Fig. 8:**
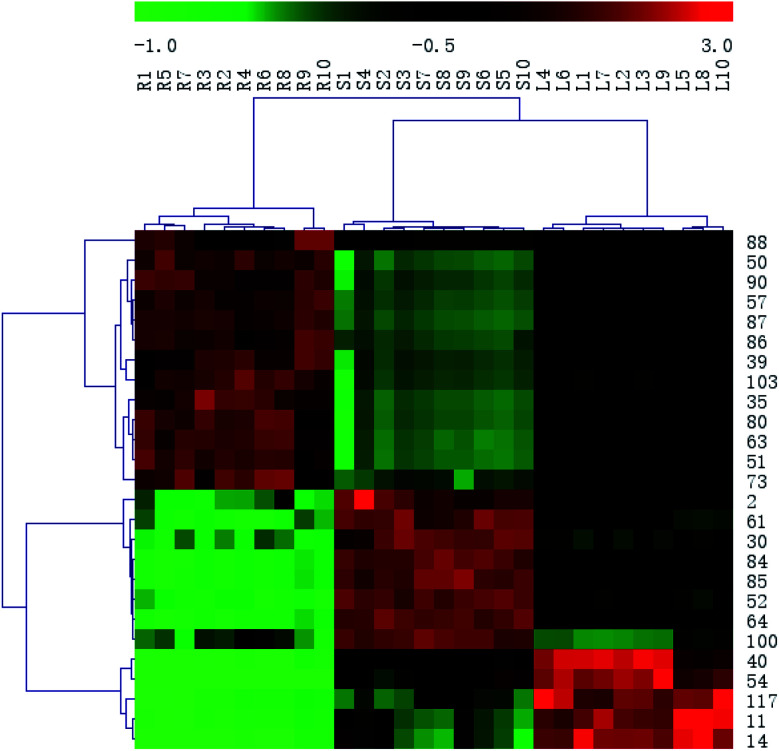
Heat-map visualizing the intensities of the potential chemical markers.

In order to systematically evaluate the chemical markers, a heat-map was generated. The hierarchical clustering heat map, intuitively visualizing the difference level of potential chemical markers in different parts, was shown in Fig. 8. The higher values were indicated by red squares, the lower values were indicated by green squares.

### 3.3. Bioactivity evaluation

#### 3.3.1 Cytotoxicity of CSE and the three parts of COT on the viability of A549 cells

**Fig. 9:**
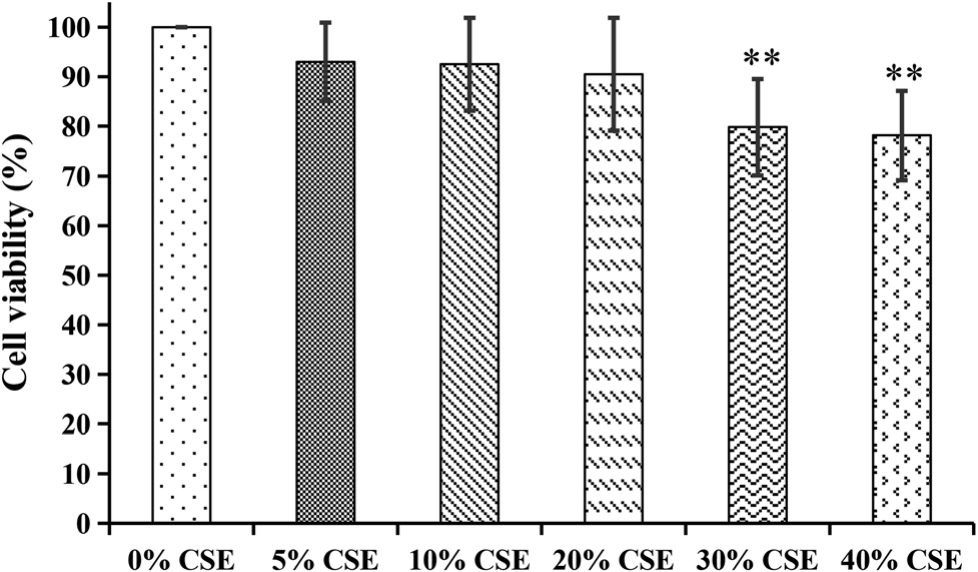
The cytotoxicity effects of different concentrations of cigarette smoke extract (CSE) on A549 cells. **p < 0.01, compared with 0% CSE group.

**Fig. 10:**
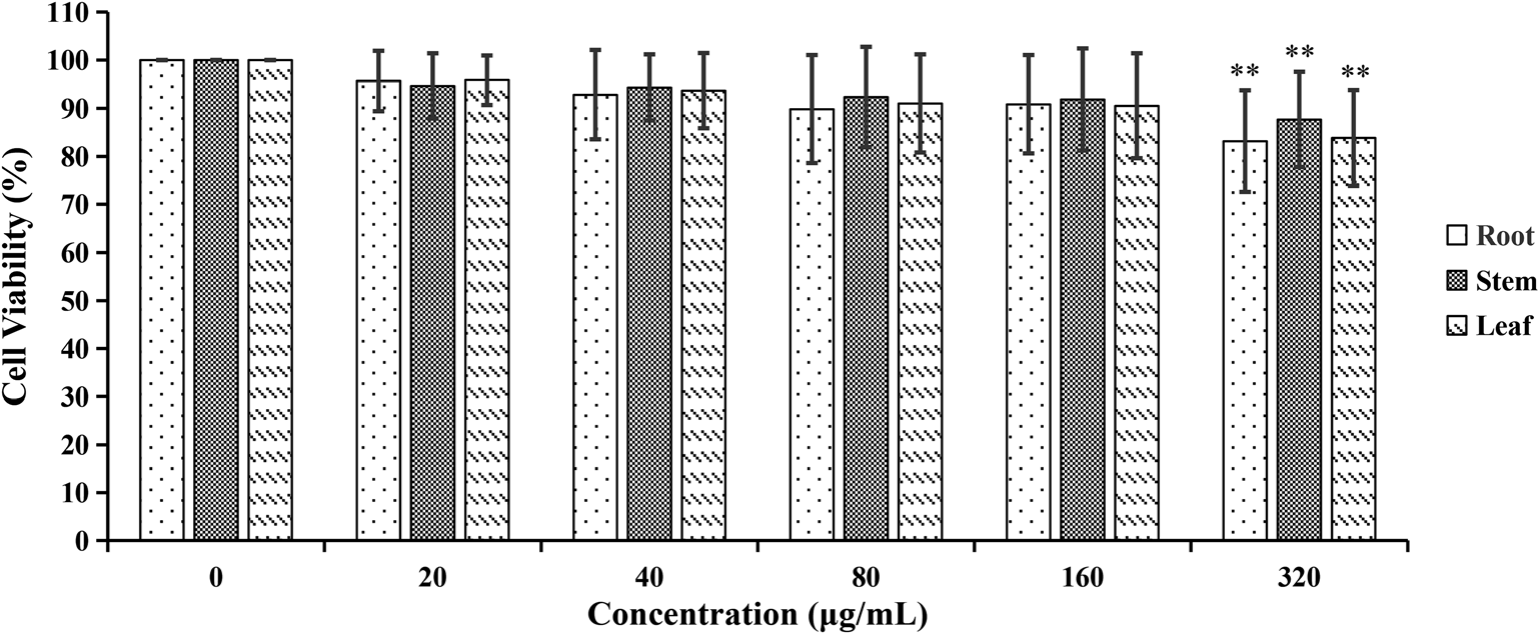
The cell viability effects of different concentrations of R_bio_, S_bio_ and L_bio_ solution on A549 cells. **p < 0.01, compared with 0 μg mL^−1^ group.

The results of MTT showed that the viability of A549 cells was obviously affected (*p* < 0.01) by 30% or 40% CSE (Fig. 9). Therefore, 20% CSE was chosen as stimulus in the following experiments. Additionally, as shown in Fig. 10, the viability of A549 cells were not significantly affected by the R_bio_, S_bio_ and L_bio_ solutions at 20–160 μg mL^−1^. So we evaluated the effects of R_bio_, S_bio_ and L_bio_ solutions at 20–160 μg mL^−1^ on CSE-stimulated A549 cells.

#### 3.3.2 Effect of root, stem and leaf of COT on CSE-stimulated pro-inflammatory cytokine levels in A549 cells

**Fig. 11:**
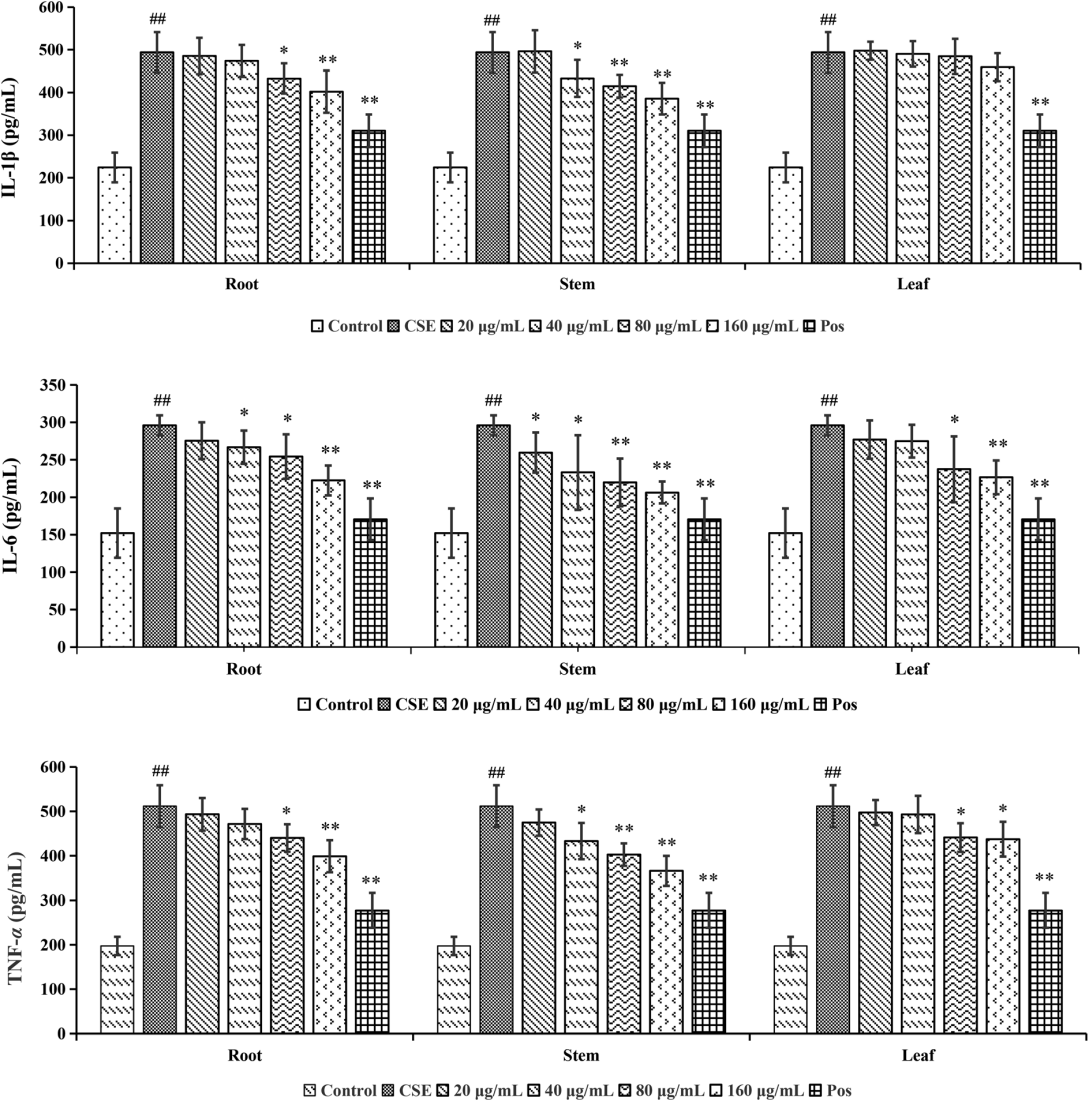
The effects of inflammatory cytokine IL-1β, IL-6 and TNF-α on root, stem and leaf (20–160 μg mL^−1^) of COT in CSE-stimulated A549 cells. ^##^*p* < 0.01, compared with control group; *p < 0.05, compared with CSE group; **p < 0.01, compared with CSE group.

The inflammatory development was characterized by the release of pro-inflammatory mediators such as interleukin-1β (IL-1β), interleukin-6 (IL-6) and tumor necrosis factor-α (TNF-α). Whether the root, stem and leaf of COT could inhibit the release of IL-1β, IL-6 and TNF-α in CSE-stimulated lung epithelial cells was investigated in this paper. As shown in Fig. 11, the production of IL-1β, IL-6 and TNF-α in A549 cells was obviously increased after treated with CSE (*p* < 0.01). However, treated with the R_bio_, S_bio_ and L_bio_ solutions could evidently decrease the levels of pro-inflammatory factors in a good dose-dependent way with a certain range of 20–160 μg mL^−1^ in CSE-stimulated cells. The R_bio_ solution could significantly decrease the levels of IL-1β, TNF-α (80 μg mL^−1^, *p* < 0.05; 160 μg mL^−1^, *p* < 0.01) and IL-6 (40 and 80 μg mL^−1^, *p* < 0.05; 160 μg mL^−1^, *p* < 0.01). The S_bio_ solution could significantly decrease the levels of IL-1β, TNF-α (40 μg mL^−1^, *p* < 0.05; 80 and 160 μg mL^−1^, *p* < 0.01) and IL-6 (20 and 40 μg mL^−1^, *p* < 0.05; 80 and 160 μg mL^−1^, *p* < 0.01). The L_bio_ solution could significantly decrease the levels of IL-6 (80 μg mL^−1^, *p* < 0.05; 160 μg mL^−1^, *p* < 0.01) and TNF-α (80 and 160 μg mL^−1^, *p* < 0.05), but showed no significantly effect on IL-1β*.* The above results showed that the S_bio_ solution had a stronger anti-inflammation effect than R_bio_ and L_bio_. It is suggested that to explore the anti-COPD effect of the COT stem *in vivo* is meaningful in further research. The different activities of root, stem and leaf of COT might be caused by the various phytochemicals in these three parts of COT. The phytochemical study showed that three parts of COT were differentiated. The bioassay study showed that three parts of COT could reduce the levels of pro-inflammatory factors to varying degrees. And the stem part had a stronger anti-COPD effect than root and leaf parts. As we all know, the material basis of different pharmacological activities is the different chemical composition. The results of the two parts of our study showed that the different activities of root, stem and leaf of COT might be caused by the various phytochemicals in these three parts of COT.

## 4. Conclusions

In conclusion, for screening analysis, a total of 120 compounds (15 shared components), including 92 from root, 56 from stem and 32 from leaf, were identified or tentatively characterized from COT. Each part of COT was rich in various kinds of structures, especially triterpeniods held the majority. For metabolomic analysis, the root, stem and leaf of COT were differentiated in both ESI^+^ and ESI^−^ modes. There were 13, 8 and 5 potential chemical markers identified from root, stem and leaf, respectively. Among the above robust markers, 5 robust chemical markers with high responses in UPLC-MS, 2 triterpenoids (pristimerin and celastrol) in root, 1 flavonoids {5,7-dihydroxy-6,8-dimethyl-3(*S*)-3-(3-methoxy-4′-hydroxybenzyl) chroman-4-one} in stem and 2 sesquiterpenoids (orbiculin I and orbiculin A) in leaf, could be used for further quality control in three parts of COT respectively. For bioassay analysis, the root, stem and leaf of COT could evidently reduce the levels of pro-inflammatory factors in a dose-dependent way within a certain range of 20–160 μg mL^−1^ in CSE-induced A549 cells. The results showed that the stem part had a stronger anti-COPD effect than root and leaf parts. The different activities might be caused by the various phytochemicals in these three parts of COT. This comprehensive phytochemical study revealed both the structural diversity of secondary metabolites and the different distributions in different parts of COT. It could provide a theoretical basis for further utilization and development of COT. And the identification of anti-COPD components from COT will be explored deeply in the future based on the current results of this study.

## Conflicts of interest

The authors declare no conflicts of interests.

## Supplementary Material
